# Crystal structures and kinetic studies of a laboratory evolved aldehyde reductase explain the dramatic shift of its new substrate specificity

**DOI:** 10.1107/S205225252300444X

**Published:** 2023-06-02

**Authors:** Shruthi Sridhar, Alberto Zavarise, Tiila-Riikka Kiema, Subhadra Dalwani, Tor Eriksson, Yannick Hajee, Thilak Reddy Enugala, Rik K. Wierenga, Mikael Widersten

**Affiliations:** a Department of Chemistry – BMC, Uppsala University, SE-751 23 Uppsala, Sweden; b Faculty of Biochemistry and Molecular Medicine, University of Oulu, PO Box 5400, Oulu FI-90014, Finland; c Biocenter Oulu, University of Oulu, PO Box 5000, Oulu FI-90014, Finland; University of Michigan, USA

**Keywords:** aldehyde reductase, enzyme functions, enzyme mechanisms, directed evolution, substrate selectivity, FucO

## Abstract

The Fe^2+^-dependent *E. coli* enzyme FucO catalyzes the reversible interconversion of short-chain (*S*)-lactaldehyde and (*S*)-1,2-propane­diol, using NADH and NAD^+^ as cofactors, respectively. Laboratory-directed evolution experiments have been carried out previously using phenyl­acetaldehyde as the substrate for screening catalytic activity with bulky substrates, which are very poorly reduced by wild-type FucO. These experiments identified the N151G/L259V double mutant (dubbed DA1472) as the most active variant with this substrate via a two-step evolutionary pathway, in which each step consisted of one point mutation. Here the crystal structures of DA1472 and its parent D93 (L259V) are reported, showing that these amino acid substitutions provide more space in the active site, though they do not cause changes in the main-chain conformation. The catalytic activity of DA1472 with the physiological substrate (*S*)-lactaldehyde and a series of substituted phenyl­acetaldehyde derivatives were systematically quantified and compared with that of wild-type as well as with the corresponding point-mutation variants (N151G and L259V). There is a 9000-fold increase in activity, when expressed as *k*_cat_/*K*_M_ values, for DA1472 compared with wild-type FucO for the phenyl­acetaldehyde substrate. The crystal structure of DA1472 complexed with a non-reactive analog of this substrate (3,4-di­meth­oxy­phenyl­acetamide) suggests the mode of binding of the bulky group of the new substrate. These combined structure–function studies therefore explain the dramatic increase in catalytic activity of the DA1472 variant for bulky aldehyde substrates. The structure comparisons also suggest why the active site in which Fe^2+^ is replaced by Zn^2+^ is not able to support catalysis.

## Abstract 2

In a laboratory-directed evolution campaign, the substrate specificity of a bacterial NADH dependent reductase (FucO) has been changed towards accepting bulky aromatic substrates via a two-step evolutionary pathway, in which each step consists of one point mutation. Enzyme kinetic data and crystal structures of the new variant (DA1472) explain the dramatic change of substrate specificity.

## 1. Introduction

**Figure 1:**
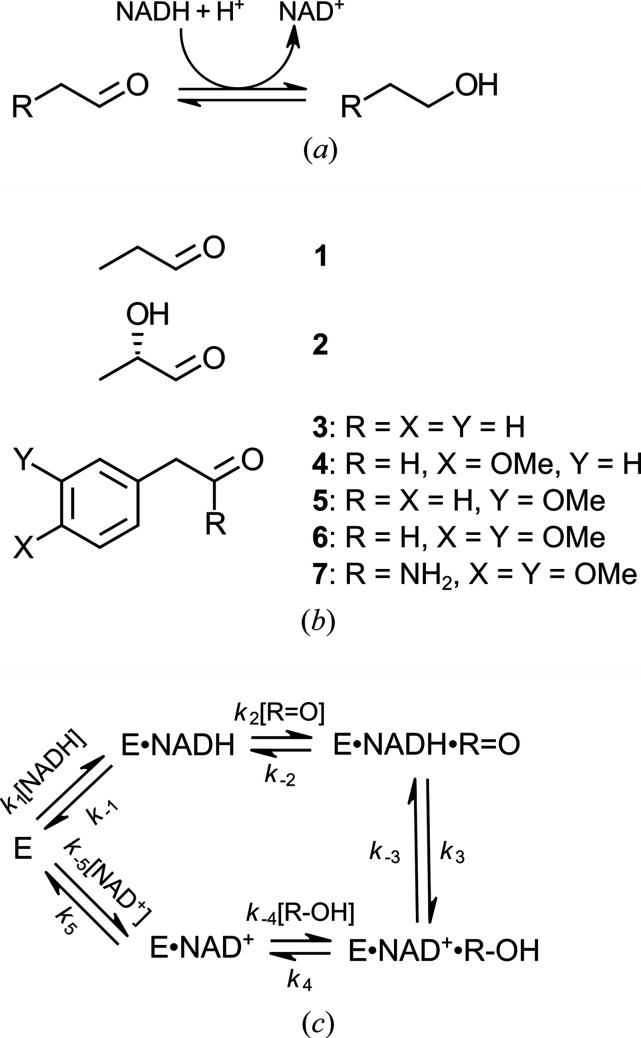
The FucO reaction and the compounds used. (*a*) FucO catalyzes the reversible aldehyde reduction/alcohol de­hydrogenase oxidation of short-chain aldehydes and their corresponding primary alcohols using NADH and NAD^+^ as cofactors, respectively. (*b*) Compounds studied as substrates and ligands. Compound **2** [(*S*)-lactaldehyde] is the physio­logical substrate. Compound **3** (phenyl­acetaldehyde) has been used in the laboratory-directed evolution experiment for screening the catalytic proficiency of variants. Compounds **4**, **5** and **6** are more bulky substrates and compound **7** (3,4-di­meth­oxy­phenyl­acetamide) is a non-reactive substrate analog of **6**. (*c*) Ordered sequential BiBi mechanism used as a model for the FucO-catalyzed aldehyde reduction.

Directed evolution is a powerful experimental tool to taylor the catalytic properties of natural enzymes to use them for applications (Savile *et al.*, 2010 ▸; Huffman *et al.*, 2019 ▸; Rosenthal *et al.*, 2022 ▸) and to create model enzymes for targeted analyses of structure–function relationships. Classic examples of such studies involve the optimization of the KEMP eliminase activity of non-natural enzymes, where subsequent crystal structure determinations show how the active sites evolve to achieve significant new catalytic activity (Khersonsky *et al.*, 2012 ▸; Blomberg *et al.*, 2013 ▸). Here it is shown which structural adaptations were introduced by directed evolution experiments that changed the catalytic properties of the *Escherichia coli* (*E. coli*) (*S*)-lactaldehyde reductase [EC 1.1.1.77 (Blikstad *et al.*, 2013 ▸, 2014 ▸)]. This enzyme, referred to as FucO, is also known as an Fe^2+^, NAD^+^-dependent group-III alcohol de­hydrogenase (Reid & Fewson, 1994 ▸) and is involved in the catabolism of rare sugars in *E. coli* (Caballero *et al.*, 1983 ▸; Baldomà & Aguilar, 1988 ▸; Blikstad & Widersten, 2010 ▸). FucO catalyzes the reversible interconversion of (*S*)-lact­aldehyde and (*S*)-1,2-propane­diol, using NADH and NAD^+^ as cofactors, respectively [Fig. 1 ▸(*a*)]. Its proposed physiological substrate is (*S*)-lactaldehyde [compound **2** in Fig. 1 ▸(*b*)], produced in the metabolism of l-fucose (Blikstad & Widersten, 2010 ▸) with the resulting (*S*)-1,2-propane­diol as the ultimate fermentation product. FucO has, by virtue of its strict regiospecificity, been of interest as a potential biocatalyst for the stereospecific oxidation of 1,2-vicinal diols into chiral α-hy­droxy­aldehydes (Blikstad *et al.*, 2013 ▸).

**Figure 2:**
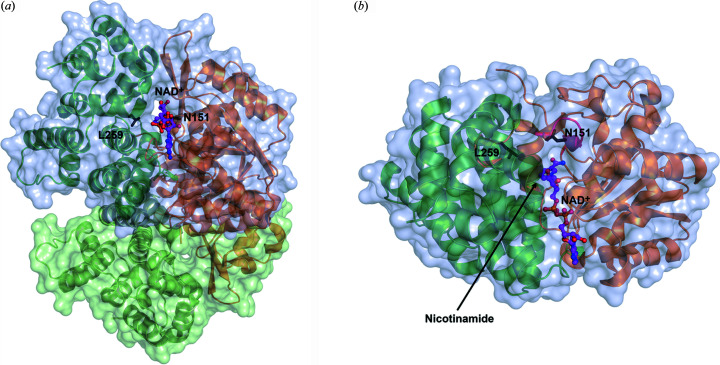
The FucO dimer. (*a*) Both subunits are in their closed conformation (PDB entry 5br4). The upper subunit is shown in side view, such that the NAD-binding groove is seen ‘end-on’. The N-terminal NAD-binding domain is brown. The C-terminal Fe^2+^-binding domain is green. The other subunit of the dimer is shown in light green. NAD^+^ (purple sticks) as bound in the groove between the two domains of the upper subunit is also shown. Asn151 (of the N-terminal NAD-binding domain) and Leu259 (of the C-terminal Fe^2+^-binding domain) are shown in black sticks. (*b*) Standard view (the NAD-binding groove is seen from above) of the upper subunit of panel (*a*), obtained by 90° rotation around the horizontal direction with respect to the view shown in panel (*a*). The catalytic site is near the nicotinamide moiety of NAD^+^ (purple sticks), which is marked by an arrow.

FucO is a dimeric enzyme consisting of two identical subunits (Fig. 2 ▸). Each subunit has two domains, the N-terminal NAD-binding domain and the C-terminal Fe^2+^-binding domain (Fig. 2 ▸). The pyrophosphate moiety of NAD is bound at the N-terminal end of the α-helix starting with Gly97. The catalytic site, near the bound nicotinamide moiety of NAD, is shaped by regions of both domains and recent structural studies have shown that the subunit can exist in both open and closed states in which the relative position of these two domains with respect to each other is different (Zavarise *et al.*, 2023 ▸). It is proposed that in the closed state the competent ternary complex is formed. In this complex the substrate and the nicotinamide moiety tightly interact with each other, such that the active site Fe^2+^ ion stabilizes the negatively charged carbonyl oxygen generated after the hydride transfer from NADH to the carbonyl carbon of the aldehyde substrate. The reduction reaction [Fig. 1 ▸(*a*)] is completed by the transfer of a proton to the carbonyl oxygen from the catalytic water, by which the alcohol product is formed.

The substrate scope of wild-type FucO is limited to short-chain aldehydes and α-hy­droxy aldehydes and their corresponding primary alcohols and vicinal diols (Blikstad & Widersten, 2010 ▸). However, an enzyme variant that efficiently catalyzes the reduction of phenyl­acetaldehyde (compound **3**) [Fig. 1 ▸(*b*)] was obtained, following iterative saturation mutagenesis of active site residues and subsequent functional selection by screening (Blikstad *et al.*, 2013 ▸, 2014 ▸). This FucO variant, dubbed DA1472, was isolated from a laboratory evolution campaign with the aim to obtain FucO variants with a broader substrate specificity, being an aldehyde reductase/alcohol de­hydrogenase capable of catalyzing the reduction of the more bulky substituted aryl-aldehydes and the oxidation of the corresponding substituted aryl­ated alcohols. Phenyl­acetaldehyde (compound **3**) was employed as the selection substrate in the directed evolution protocol. Its use was motivated by the notion that the wild-type enzyme displays measurable, albeit very low, catalytic activity with this aldehyde (Table 2) (Blikstad *et al.*, 2013 ▸), thus demonstrating catalytic competence, but leaving ample room for improvement. Also, the thermodynamic drive of the reductive reaction direction increased the sensitivity of the screening.

The isolated DA1472 variant is a double point mutation variant, N151G and L259V. It exhibits profoundly shifted substrate selectivity, compared with the wild-type FucO, and the aim of the current study was to extend the available kinetic data (Blikstad *et al.*, 2013 ▸, 2014 ▸) of the DA1472 variant as well as of its parents, the D93 (L259V) and the A5 (N151G) variants with a systematic determination of their *k*_cat_ and *K*_m_ values for a range of aldehyde substrates. Furthermore, we have complemented these enzyme kinetic studies with X-ray crystallography of the DA1472 and D93 variants to obtain a comprehensive description of the structure–function relationship of the new DA1472 variant. Four new structures are reported including structures of the DA1472 variant complexed with a non-reactive analog of the bulky phenyl­acetaldehyde substrate.

## 2. Materials and methods

### 2.1. Chemicals, reagents and molecular biology components

Buffer components and other bulk chemicals were of the highest purity commercially available. Propanal (compound **1**, Fig. 1 ▸; ≥98%, Sigma–Aldrich W292312), (*S*)-lactaldehyde (compound **2**; 1 *M* solution in H_2_O, Sigma–Aldrich 47014) and 3,4-di­meth­oxy­phenyl­acetamide (compound **7**, Sigma–Aldrich CDS011402) were used in assays and reaction mixtures as provided by the manufacturer. Phenyl­acetaldehyde (compound **3**, ≥95%, Sigma–Aldrich W287407) was column purified before use. The 4-meth­oxy-, 3-meth­oxy- and 3,4-di­meth­oxy-substituted aldehydes (compounds **4**−**6**) were synthesized and purified as described (Al-Smadi *et al.*, 2018 ▸).

### 2.2. Protein expression and purification.

The wild-type enzyme and its variants were expressed and purified as described previously (Blikstad & Widersten, 2010 ▸).

### 2.3. Crystallization, structure determination and structure refinement.

The crystals were obtained by cocrystallization at room temperature, using the sitting-drop vapor diffusion method. The results of the crystallization experiments were monitored by the *IceBear* research data-management software (Daniel *et al.*, 2021 ▸). The details of the crystallization and crystal treatment protocols are described in Table S1 of the supporting information. In all cases, the datasets were collected from frozen crystals. The data collection and data-processing methods are listed in Table 1 ▸. The structures were solved by molecular replacement using the expert mode of *PHASER* (McCoy *et al.*, 2007 ▸) as implemented in *CCP4i2* (Potterton *et al.*, 2018 ▸) by splitting the search molecule (PDB entry 1rrm; unpublished work) into two parts, consisting of residues 1–185 and 186–383. In all structures there was one dimer per asymmetric unit. The structures were refined using alternately *REFMAC5* (Kovalevskiy *et al.*, 2018 ▸) for refinement calculations and *Coot* (Casañal *et al.*, 2020 ▸) for manual improvement of the protein structure by inspecting the corresponding electron density maps, for identifying water molecules and for building ligands. The quality of the structures was checked using the validation tools of *Coot* and PDB validation reports. The final refinement statistics are listed in Table 1 ▸. In the complexes obtained by cocrystalization with the nucleotides NADH or NAD^+^, the density of the ADP-ribose part of the nucleotide was always clearly defined by the electron density map, whereas the electron density for the nicotinamide moiety was usually weaker, suggesting that either the nicotinamide ring is partially disordered, or NAD^+^ or NADH is partially hydrolysed, or both. In the structures with poor density for the nicotinamide ring the nucleotide was modeled as ADP-ribose (ADPR), as also described previously (Zavarise *et al.*, 2023 ▸).

The dataset of the binary complex of D93 (L259V) and NADH (PDB entry 7qlg) was collected at beamline I24 of the Diamond Light Source (DLS, Oxford, UK), and *XDS* (Kabsch, 2010 ▸; Sparta *et al.*, 2016 ▸) was used for data processing at a resolution of 2 Å. The merged intensities were obtained using *AIMLESS* (Evans & Murshudov, 2013 ▸). The nucleotide ligand was built as NADH, with the ligand name NAI.

The dataset of the binary complex of FucO DA1472 (N151G/L259V) and NADH (PDB entry 7qnh) was collected using the Bruker Microstar X8 diffractometer home X-ray source, integrated using *SAINT* and scaled using *SADABS* of the *PROTEUM3* suite. The unmerged intensity dataset, provided by *XPREP*, with a resolution limit of 2.2 Å, was subsequently input to the *CCP4i2* X-ray data-reduction and analysis pipeline and merged with *AIMLESS*. TLS refinement was used to improve the structure. The TLS groups were chosen manually, following the domain structure of FucO, such that two groups were identified; the N-terminal domain for TLS group 1 (residues 1–185) and the C-terminal domain for TLS group 2 (residues 186–383). The fit of NADH in the corresponding electron density map is shown in Fig. S1 of the supporting information.

The datasets of the two ternary complexes of DA1472 (N151G/L259V), obtained by cocrystallization with compound **7** and NAD^+^ or NADH (PDB entries 7qlq and 7qls, respectively) were collected at the BioMAX beamline of MAX IV (Lund, Sweden). The datasets of these crystals were processed with *DIALS* (Winter *et al.*, 2018 ▸) and *XDS* respectively, and scaled and merged using *AIMLESS*. Towards the end of the refinement, TLS and NCS refinements were used to improve the structure further, using the same TLS groups as outlined above. In both these structures the bound ligands in the active site are the substrate analog (compound **7**) and ADPR, with the ligand names E9I and APR, respectively. The restraints of the model of the substrate analog, as used in the refinement calculations, were obtained from the Global Grade Server (https://grade.globalphasing.org/cgi-bin/grade/server.cgi). The fit of this ligand in the corresponding electron density map is shown in Fig. S1.

### 2.4. Structure analysis

The four new structures were compared with each other and with three FucO structures (available in the PDB), with a wild-type catalytic site, as well as with two previously described structures of point-mutated FucO variants (PDB entries 7qnf and 7r0p; Zavarise *et al.*, 2023 ▸). The structures used to study the wild-type active site are the 1rrm structure (PDB entry 1rrm), refined at 1.60 Å resolution, complexed with Zn^2+^ and ADPR; the 5br4 structure (PDB entry 5br4; Cahn *et al.*, 2016 ▸), refined at 0.91 Å resolution, and complexed with Zn^2+^ and NAD^+^; as well as the 2bl4 structure (PDB entry 2bl4; Montella *et al.*, 2005 ▸) refined at 2.85 Å, complexed with Fe^2+^ and NAD^+^. The 1rrm and 2bl4 structures have the wild-type sequence, whereas the 5br4 structure involves a point-mutation variant in which a residue at the binding site of the adenine moiety of NAD^+^ (far from the catalytic site) was mutated. This mutation causes only minor, local structural changes. The conformations of the main chain traces of the 1rrm, 5br4 and 2bl4 structures around the catalytic site are the same.

The two previously reported structures of point-mutated FucO variants used for comparison are the structures of the double mutant N151G/L259 (PDB entry 7qnf, refined at 2.14 Å resolution, complexed with Fe^2+^) as well as the D47 variant, which has the F254I point mutation (PDB entry 7r0p, refined at 1.48 Å resolution, complexed with Fe^2+^). In the latter two structures, the active sites are also complexed with a nucleotide, ADP-ribose and NAD^+^. The active site of subunit *B* of the 7qnf structure is also complexed with the substrate ethyl­eneglycol, interacting directly with the active site Fe^2+^ ion via one of its oxygen atoms. Using the D47 variant the kinetic and structural properties of the F254I point mutation have been probed. Like for D93, the catalytic properties of D47 for the oxidation of propanol-1 have not changed very much with respect to the wild-type (Blikstad *et al.*, 2014 ▸), and there are only minor structural rearrangements of the main chain for residues 253–254–255, being located near the edge of the substrate specificity pocket.

In the four new crystal structures described here, the bound active site nucleotide is NADH (PDB entries 7qnh, 7qlg) or ADPR, and compound **7** (PDB entries 7qls, 7qlq) and each of these active sites are also complexed with Fe^2+^. In each of these four structures, FucO was crystallized with a dimer in the asymmetric unit and in these structures the two domains of each subunit adopt the closed conformation. For structural comparisons the subunits were superimposed on each other with the SSM protocol (Krissinel & Henrick, 2004 ▸), as implemented in *Coot*. The figures, which visualize the structural information, were made using *PyMOL* (*The PyMOL Molecular Graphics System*, Version 2.3, Schrödinger, LLC).

### 2.5. Steady-state kinetics

Aldehyde reduction was assayed spectrophotometrically by the time-dependent concomitant oxidation of NADH, followed at 340 nm in a 0.5 cm cuvette in a 0.1 *M* sodium phosphate buffer, pH 7.5 at 30°C. The given concentrations of the aldehyde substrates included in the activity assays refer to the sum of the carbonyl and hydrated forms of the aldehydes. The Michaelis–Menten model [equation (1)] or a reparametrized version thereof [equation (2)] was fitted to the initial rates to extract steady-state parameters, using the programs *MMFIT* or *RFFIT*, respectively, of the *Simfit* package (https://www.simfit.org.uk). In cases where substrate inhibition was observed (*e.g.* see Fig. S2), a model including reversible formation of a dead-end complex [equation (3)] was fitted using the *QNFIT* program of the *Simfit* package. The parameter *K*_IS_ is the equilibrium dissociation constant of the non-productively bound aldehyde substrate.

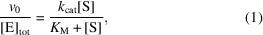




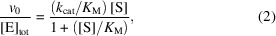




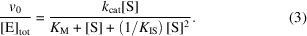

Due to the practical limitations of the spectrophotometric assay, for example a high starting absorbance of NADH which limits the maximal concentration that can be added to the assay mixtures, and the limited solubility of the tested aldehyde substrates, the non-varied substrate could not be present at saturating concentrations in all cases. Pseudo-first-order reaction conditions could therefore not be fully met in these cases. Hence, values of *k*_cat_ and *k*_cat_/*K*_M_ should, in noted cases (see Table 2 ▸ footnotes), be considered as lower estimates. NADH concentration was kept at 0.4 m*M*, which corresponds to 20 × *K*_M_ for the wild-type and A5 enzymes, 5 × *K*_M_ for D93 and 0.62 × *K*_M_ for DA1472. Aldehydes were added at concentrations of 2–50 m*M* (compounds **1** and **2**), 0.1–4.5 m*M* (compound **3**), 0.05–1 m*M* (compound **4**), 0.025–0.75 m*M* (compound **5**) and 0.025–1.5 m*M* (compound **6**). Kinetic parameters were also determined in the presence of varying concentrations of NADH (20–800 µ*M*) and either 30 m*M* (wild-type, 14 × *K*_M_ and DA1472, 2.1 × *K*_M_) or 50 m*M* (A5, 4.6 × *K*_M_ and D93, 17 × *K*_M_) of aldehyde **1**. The ratios of the aldehydic versus the hydrated states of the aldehyde substrates were not determined.

### 2.6. pH dependency of the DA1472 catalyzed reduction

The reduction rates of compound **3**, expressed as *k*_cat_ or *k*_cat_/*K*_M_, were determined at pH values between 6–8, as described above, in 0.1 *M* sodium phosphate buffers, in the presence of 0.4 m*M* NADH and at 30°C.

### 2.7. Solvent viscosity effect

Initial velocities of the DA1472 catalyzed reduction of 0.02–0.75 m*M* compound **5** were recorded in 0−30%(*w*/*v*) sucrose in 0.1 *M* sodium phosphate buffer, pH 7.0 at 30°C. Translation of sucrose concentrations into relative viscosity was carried out as described previously (Blikstad & Widersten, 2010 ▸). The degree of dependency of *k*_cat_ and *k*_cat_/*K*_M_ on the relative viscosity (η_rel_) was estimated by fitting a linear model using *LINFIT* in the *Simfit* package [Table S2, Fig. S2(*c*)].

## 3. Results and discussion

### 3.1. Enzyme kinetic properties of the DA1472 variant and its D93 and A5 precursors

The DA1472 variant emerged from two separate but converging evolutionary pathways (Blikstad *et al.*, 2013 ▸, 2014 ▸). One route produced the single mutant L259V (dubbed variant D93). D93 displays features of a ‘generalist’ enzyme regarding substrate selectivity, retaining reasonable activities with compound **1** and its physiological substrate compound **2** (Table 2 ▸) and, in addition, being able to catalyze the reduction of the more bulky compound **3** with 13-fold improved catalytic efficiency (*k*_cat_ */K*_M_) compared with the wild-type (Table 2 ▸). In a parallel search, FucO variant A5 (N151G) was isolated. A5 catalyzes the reduction of its physiological substrate (compound **2**) with a similar *k*_cat_ value as the wild-type, but it requires higher substrate concentrations to reach saturation (*K*_M_ of compound **2** has increased 100-fold; Table 2 ▸). The substantial increase in *K*_M_ for compound **2** reflects the importance of the acetamide side chain of Asn151, being hydrogen bonded with the 2-hydroxyl moiety of compound **2** (Zavarise *et al.*, 2023 ▸). However, the activity with the more bulky substrate, compound **3**, as judged by *k*_cat_/*K*_M_, has increased by 150-fold with respect to the wild-type (Table 2 ▸).

The double mutant (DA1472) was subsequently isolated from a second-generation library parented by the D93 variant. The observed improvement in activity with compound **3** of DA1472 requires both substitutions to be present (Table 2 ▸). Comparing the activities of A5 and D93 with those of DA1472, it is clear that DA1472 is closer to A5 in the activity profile, demonstrating the important contribution of the N151G substitution to the shift in substrate selectivity, although combined epistatic effects can be observed. For example, the substantially lower *K*_M_ of compound **3** shown by DA1472 (approximately ninefold, compared with the wild-type, Table 2 ▸) is not the result of step-wise additive effects by the individual mutations, but rather depends on the combination of substitutions.

In order to further analyze the substrate scope of DA1472 and its parent variants, regarding aryl­ated aldehydes and to assess possible effects on the physiological reaction caused by the introduced mutations, an extended set of phenyl­acetaldehyde derivatives [compounds **4**, **5** and **6**; Fig. 1 ▸(*b*)] were also investigated as substrates (Table 2 ▸). The results illustrate a dramatic shift in substrate scope. The preference of the wild-type enzyme for short-chain aliphatic 2-hy­droxy­aldehydes has been shifted to favor aryl-substituted, non-hydroxy­lated aldehydes. *k*_cat_/*K*_M_ for phenyl­acetaldehyde (compound **3**) has increased approximately 9000-fold whereas the activity with the native (*S*)-lactaldehyde (compound **2**) has decreased approximately 80-fold (Table 2 ▸). Furthermore, the accentuated (*S*)-selectivity of wild-type FucO in the oxidation of 1,2-propane­diol [320-fold in favor of (*S*)-1,2-propane­diol over the (*R*)-enantiomer] is lost in this variant (Blikstad *et al.*, 2014 ▸). The activities displayed by DA1472 with the meth­oxy-substituted aldehydes **4**, **5** and **6** are notably high and the *k*_cat_/*K*_M_ values for the monosubstituted compounds **4** and **5** even exceed that of the wild-type enzyme with the physio­logical substrate **2** (Table 2 ▸). Different degrees of substrate inhibition were observed in the reactions with all phenyl­acetaldehyde derivatives and a model incorporating the reversible formation of a dead-end quaternary complex [equation (3)] was fitted to the steady-state initial rates. An example saturation curve of the DA1472 catalyzed reduction of compound **3** is shown in Fig. S2, together with the proposed reaction scheme. As judged by the estimated values of the apparent dissociation constants of these proposed dead-end complexes (*K*_IS_ in Table 2 ▸), the *para*-meth­oxy substituted **4** and **6** appear to be less prone to bind in a non-productive mode.

### 3.2. The crystal structures of the D93 and DA1472 variants

High-resolution crystal structures were obtained of the D93 (L259V) and the DA1472 (N151G/L259V) variants cocrystallized with NADH (at 2.0 and 2.2 Å resolution, respectively, Table 1 ▸). NADH is bound in the groove shaped by the N-terminal NAD-binding domain and the C-terminal Fe^2+^-binding domain (Fig. 2 ▸). The mode of binding of NADH in the active site, including the nicotinamide moiety, is well defined by the electron density map (Fig. S1), being the same in both structures. Also, crystal structures were obtained of the DA1472 variant in the presence of the bulky substrate analog, compound **7**, cocrystallized in the presence of either NAD^+^ or NADH (at 2.6 and 2.4 Å resolution, respectively, Table 1 ▸). In the structures of the latter two complexes, the mode of binding of the nicotinamide moieties of NAD^+^ and NADH is not defined by the electron density map and therefore, the nucleotide was modeled as ADP-ribose (ADPR, as described in the Materials and methods[Sec sec2]). The mode of binding of the substrate analog is the same in both structures and well defined by the electron density map (Fig. S1).

**Figure 3:**
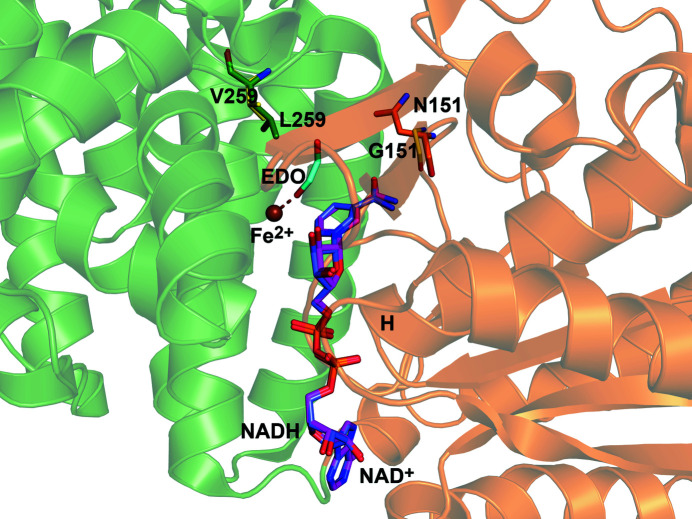
The two amino acid substitutions of the DA1472 variant make the substrate specificity pocket wider. In this figure, the mode of binding of NADH (magenta sticks) and Fe^2+^ to the active site of the DA1472 variant are shown, as well as (in yellow) the residues Gly151 and Val259 (PDB entry 7qnh). Also shown are the superimposed NAD^+^ (gray sticks), as well as the residues Asn151 (orange, from the NAD-binding domain) and Leu259 (green, from the Fe^2+^-binding domain) of the wild-type active site (PDB entry 5br4). The mode of binding of the product of the reduction reaction, ethyl­eneglycol (EDO, cyan), which interacts with its oxygen atom with the Fe^2+^ ion (PDB entry 7qnf), as highlighted by a dotted line. ‘H’ identifies the N-terminal end of the pyrophosphate-binding helix of the NAD-binding domain (brown).

The structures of the protein regions with the mutated residues are well defined by the electron density maps and the conformations of these regions are the same as in the wild-type. The comparison of the structures of the active sites of the wild-type and the DA1472 variant shows that the shorter side chains of the L259V (valine instead of leucine) and N151G (glycine instead of asparagine) amino acid substitutions provide more space in the region where the substrate binds (Figs. 3 ▸ and 4 ▸). The substrate-binding region is identified by the mode of binding of the substrate ethyl­eneglycol, as observed in PDB entry 7qnf, also shown in Figs. 3 ▸ and 4 ▸. Fig. 3 ▸ also includes the mode of binding of NADH to the active site of the DA1472 variant (PDB entry 7qnh). This is the same as observed for NAD^+^ bound in the wild-type active site (PDB entry 5br4; Fig. 3 ▸).

**Figure 4:**
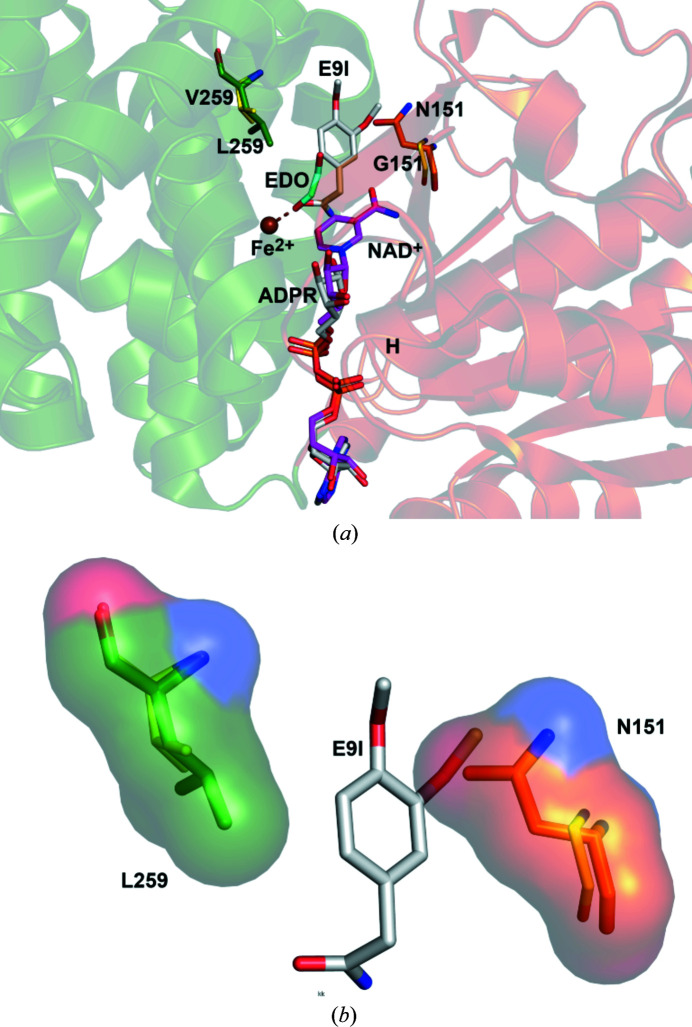
The mode of binding of compound **7** (3,4-di­meth­oxy­phenyl­acetamide) in the wider substrate specificity pocket of the DA1472 variant. (*a*) Shown are the substrate analog of 3,4-di­meth­oxy­phenyl­acetamide (gray, E9I), as well as the Fe^2+^ ion, ADPR and the residues Gly151 and Val259 (PDB entry 7qls). Superimposed are NAD^+^ and the residues Asn151 and Leu259 of the wild-type active site (PDB entry 5br4). Also shown is the superimposed ethyl­eneglycol (EDO, cyan) as observed in the DA1472 variant (PDB entry 7qnf) and interacting with the Fe^2+^ ion (dotted line). ‘H’ identifies the N-terminal end of the pyrophosphate-binding helix of the NAD-binding domain (brown). The Fe^2+^-binding domain is colored green. (*b*) Zoomed-in view of the substrate-binding pocket with bound 3,4-di­meth­oxy­phenyl­acetamide, visualizing the extra space generated by the N151G and the L259V amino acid changes. Gly151 (yellow) and Val259 (yellow) are of the DA1472 variant of the structure with bound 3,4-di­meth­oxy­phenyl­acetamide (gray, E9I) (PDB entry 7qls). Superimposed are Asn151 (orange) and Leu259 (green) of the wild-type active site (PDB entry 5br4), which are also represented by their molecular surfaces.

**Figure 5:**
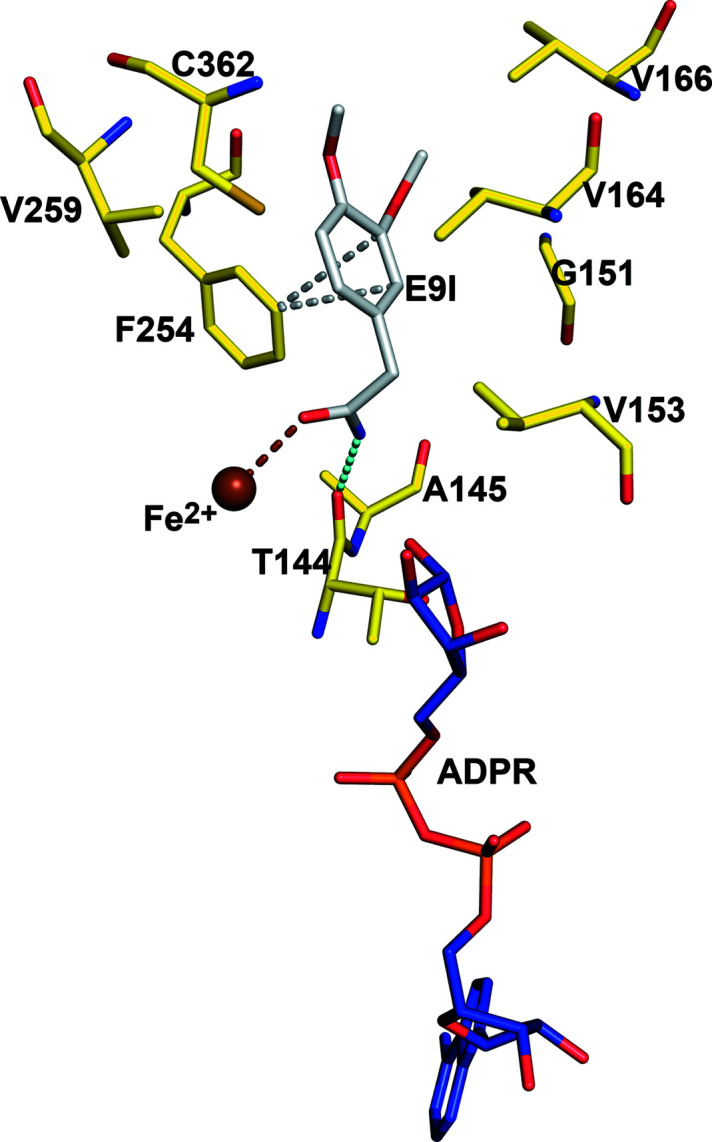
Interactions of the bulky 3,4-di­meth­oxy­phenyl­acetamide substrate analog in the wider substrate specificity pocket of the DA1472 variant. The edge-to-face interaction between the aromatic rings of Phe254 and the substrate analog (gray, E9I) is highlighted by dotted lines, identifying contact distances shorter than 3.8 Å (PDB entry 7qls). Also shown are the ADPR molecule and the Fe^2+^ ion. The interactions of the oxygen and the nitro­gen atoms of the amide moiety of the substrate analog with Fe^2+^ and O(Thr144), respectively, are also highlighted by dotted lines. The side chains of Phe254, Val259 and Cys362 (left of the C-terminal domain); and Val153, Val164 and Val166 (right of the N-terminal domain) line the substrate specificity pocket.

The mode of binding of compound **7** (3,4-di­meth­oxy­phenyl­acetamide) as provided by the structures obtained from the cocrystallization experiments in the presence of NADH or NAD^+^, is shown in Fig. 4 ▸. The observed mode of binding of compound **7** is possible because of the N151G and L259V amino acid changes of DA1472. In particular, the side chain of Asn151 of the wild-type active site clashes with the mode of binding of compound **7** (Fig. 4 ▸). The oxygen atom of the amide group of compound **7** interacts with the Fe^2+^ ion and the amide nitro­gen is hydrogen bonded to O (Thr144) (Fig. 5 ▸). The phenyl ring of compound **7** fits in a hydro­phobic pocket and has a presumably favorable (Serrano *et al.*, 1991 ▸; Chatterjee *et al.*, 2019 ▸; Chakrabarti & Bhattacharyya, 2007 ▸) edge-to-face interaction with the phenyl ring of Phe254 (Fig. 5 ▸). The two amino acid changes allow the more bulky phenyl­acetaldehyde substrates (compounds **3**–**6**) to be efficiently reduced (Table 2 ▸) by the DA1472 variant. The structures of the DA1472 variant complexed with this substrate analog (Figs. 4 ▸ and 5 ▸) suggest the mode of binding and the favorable interactions of the aromatic phenyl­acetaldehyde substrates in the active site of this FucO variant.

### 3.3. Structural enzymology properties of the DA1472 variant

Two other enzymological properties of the DA1472 variant were investigated: the dependency of the kinetic properties on medium viscosity as well as the pH dependency of the reduction reaction. The viscosity of the solvent affects the kinetic rates of diffusion-controlled steps, including association with the substrate (in the case of diffusion-controlled reactions), as well as the dissociation of product(s) and/or conformational changes that are rate limiting (Schurr, 1970 ▸; Demchenko *et al.*, 1989 ▸; Eser & Fitzpatrick, 2010 ▸). The dependency on viscosity was tested with the *meta*-substituted compound **5** as the aldehyde substrate, showing complete (unit) dependency for *k*_cat_ and *k*_cat_/*K*_M_ [Table S2, Fig. S2(*c*)]. These properties are in line with rate-limiting product release, presumably associated with conformational changes, as was also proposed for the wild-type enzyme, when using propanal (compound **1**) as the aldehyde substrate (Blikstad & Wider­sten, 2010 ▸). The pH dependency of *k*_cat_ and *k*_cat_/*K*_M_ in the reduction of compound **3** is relatively flat (Fig. S3), as observed for the wild-type enzyme for the reduction of compound **1** (Blikstad & Widersten, 2010 ▸), which is in agreement with the proposed reaction mechanism, whereby a catalytic water molecule provides the proton for the reduction reaction (Zavarise *et al.*, 2023 ▸).

**Figure 6:**
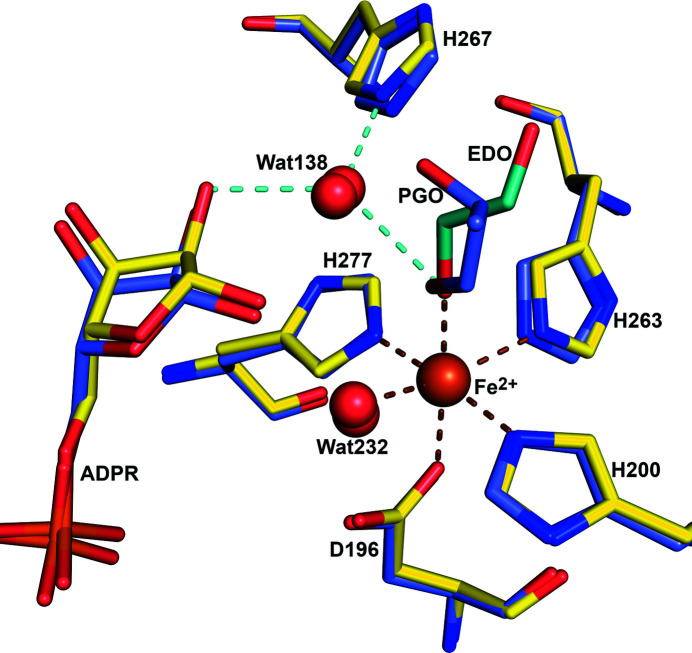
The architecture of the catalytic site of the DA1472 variant and wild-type FucO is the same. The catalytic site of the DA1472 variant [PDB entry 7qnf, complexed with ethyl­eneglycol (EDO, cyan), Fe^2+^ and ADPR] is shown with yellow carbons and the superimposed active site of wild-type FucO [PDB entry 1rrm, complexed with ADPR and Zn^2+^, as well as S-(1,2)-propane­diol (PGO)] is shown with blue carbons. The Fe^2+^ ion of the DA1472 variant and the Zn^2+^ ion (not visible) of the wild-type superimpose exactly. The Fe^2+^ ion is coordinated by interactions with the side chains of Asp196, His200, His263 and His277 and with a water (Wat232) as well as an oxygen atom of the substrate ethyl­eneglycol. The latter interactions are highlighted by dotted lines. The oxygen atom of ethyl­eneglycol (EDO) of the 7qnf structure is replaced by an oxygen atom of (S)-1,2-propane­diol (PGO) in the 1rrm structure, whereas waters Wat232 and Wat138 correspond to waters bound in overlapping sites of the 1rrm structure. Wat138 is the catalytic water, which is hydrogen bonded to EDO, to the ribose moiety of ADPR and to NE2(His267) (dotted lines).

Structure comparisons suggest that the geometry of the DA1472 catalytic site is also the same as seen for the wild-type. For example, the superpositioning of the structures of DA1472 (7qnf, complexed with ethyl­eneglycol, refined at 2.14 Å) and the high-resolution structure of wild-type FucO (PDB entry 1rrm, chain A, complexed with (*S*)-1,2-propane­diol, refined at 1.6 Å) shows no structural differences (Fig. 6 ▸). In these two structures, the modeled nucleotide is ADPR and in both active sites a substrate is bound. In the active site of DA1472 the metal ion is Fe^2+^, whereas in the 1rrm structure the bound metal ion is Zn^2+^. The enzyme is only active in the presence of Fe^2+^ and is inactivated in the presence of Zn^2+^, Cu^2+^ and Cd^2+^ (Montella *et al.*, 2005 ▸). The Fe^2+^ ion is coordinated by a carboxyl­ate oxygen of the Asp196 side chain and the NE2 nitro­gen atoms of His200, His263 and His277. In the 7qnf structure the Fe^2+^ ion is also coordinated by an additional water (WAT232) as well as an oxygen atom of the substrate (Fig. 6 ▸). The latter oxygen atom is replaced by a water in the case where there is no substrate, substrate analog or nicotin­amide moiety bound in the active site. This coordination geometry has also been observed in the structures of the unliganded active sites of homologous de­hydrogenases, such as the bacterial alcohol de­hydrogenase of *Zymomonas mobilis* (Moon *et al.*, 2011 ▸) and *Klebsiella pneumonia* (Marçal *et al.*, 2009 ▸).

**Figure 7:**
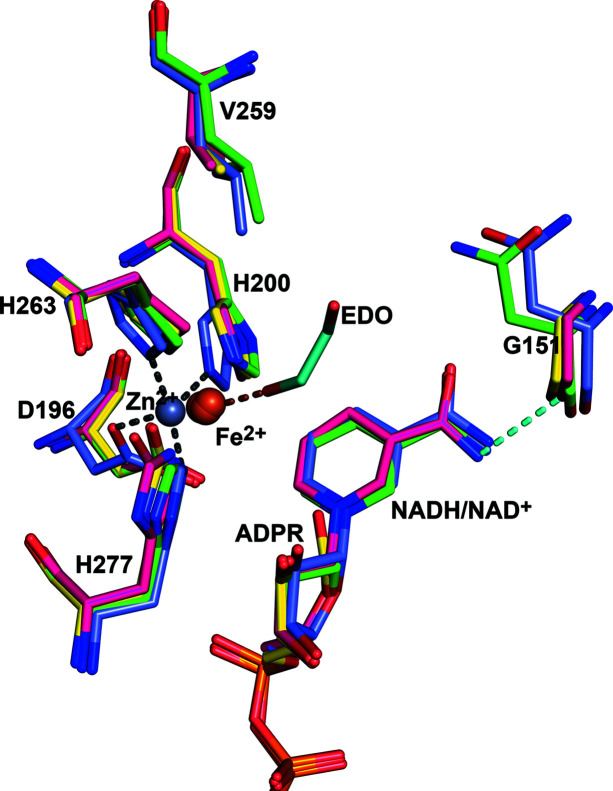
The modes of binding of NADH and NAD^+^ in the FucO catalytic site are the same. The superimposed structures are of the DA1472 variant (magenta, PDB entry 7qnh) with bound NADH and of the D47 variant (light-green, PDB entry 7r0p) with bound NAD^+^. Both active sites are also complexed with Fe^2+^. Also included are the superimposed wild-type catalytic site of the 5br4 structure (blue, with bound NAD^+^ and Zn^2+^) and the superimposed active site of the 7qnf structure of the DA1472 variant (yellow), with the bound ethyl­eneglycol (EDO, cyan), ADPR and Fe^2+^. The Zn^2+^ position (as captured in the 5br4 structure) is shifted by approximately 1 Å, away from the canonical Fe^2+^ position. The interactions of the Zn^2+^ ion with the side-chain atoms of Asp196, His200, His263 and His277 in the 5br4 structure, and the interaction of the Fe^2+^ ion with EDO in the 7qnf structure, are highlighted by dotted lines. The hydrogen bond between the amide nitro­gen of the nicotinamide moiety of NAD^+^ and NADH and the peptide oxygen of residue 151 is also highlighted by a dotted line.

In the FucO structures of the point-mutation variants reported here, as well as in previous work (Zavarise *et al.*, 2023 ▸), the active sites are complexed with Fe^2+^, like in the wild-type 2bl4 structure (Montella *et al.*, 2005 ▸), and the Fe^2+^ position is the same in all these structures. In the 1rrm and 5br4 crystal structures this Fe^2+^ is replaced by Zn^2+^. The Fe^2+^ ion interacts with the reactive oxygen atom of the substrate (Fig. 6 ▸). This interaction stabilizes the intermediate of the reaction cycle that has the negatively charged oxyanion, generated in the reduction by the transfer of the hydride ion from NADH to the carbonyl carbon atom of the substrate. In the FucO active site, the Fe^2+^ ion tightly interacts with the nicotinamide moiety, as the distance between the Fe^2+^ ion and the C5 atom of the nicotinamide group is about 3 Å. The Fe^2+^ position is the same in the presence of bound ADPR, NADH or NAD^+^ (Fig. 7 ▸). The position of the nicotinamide moieties of the latter two structures is well defined by the corresponding electron density maps (Fig. S1), being hydrogen bonded to residues of the NAD-binding domain. The oxygen atom of the amide group of the nicotinamide part of NAD is hydrogen bonded to OG1 of Thr149, whereas its nitro­gen atom is hydrogen bonded to the peptide oxygen atom of residue 151 (Fig. 7 ▸) and a carboxyl­ate side-chain oxygen atom of Asp102. Further studies are required to understand why, in the presence of Zn^2+^ (instead of Fe^2+^), the enzyme is inactive; but in this respect, it is of interest to note that in the Zn^2+^ active site, complexed with NAD^+^ (as captured in the atomic resolution 5br4 structure), the Zn^2+^ mode of binding is shifted significantly away from the canonical Fe^2+^ position (Fig. 7 ▸), such that the distance between the Zn^2+^ ion and the C5 atom of the nicotinamide is 4 Å (instead of 3 Å). Also, the side chains of Asp196, His200 and His263, which interact with the Zn^2+^ ion of the 5br4 structure, have shifted somewhat in the same direction as the Zn^2+^ ion (Fig. 7 ▸). In the Fe^2+^-complexed active sites such a shift is not observed in any of the available structures. These structural differences between the Zn^2+^/NAD^+^- and the Fe^2+^/NAD^+^-complexed active sites could be related to the notion that FucO is inactive in the presence of Zn^2+^.

The 30-fold increase in *K*_M_ for NADH displayed by DA1472 (but not by A5 and D93; Table 2 ▸) cannot be explained directly from the available structures of the complexes with bound NAD^+^ or NADH, which suggest that the amino acid changes do not affect the mode of binding of the nucleotide. It is proposed that in solution FucO exists in both open and closed states (Zavarise *et al.*, 2023 ▸). Indeed, it has been demonstrated that group-I alcohol de­hydrogenases exhibit such dynamic nucleotide binding behavior (Plapp, 2010 ▸). The existence of closed/open states of the binary E·NAD^+^(H) complexes of the FucO enzyme, where one of the conformations would exhibit lower affinity for the coenzyme, can possibly provide an explanation to why the mutations in DA1472 result in this behavior. If the substitutions affect the relative stabilities of such states, then an apparent dissociation constant such as *K*_M_ could be affected as a consequence. This hypothesis is in line with current theories on the functional evolution of enzymes which state that conformational sub-states and their relative stabilities can form the basis for a given functional profile (James & Tawfik, 2003 ▸; Tokuriki & Tawfik, 2009 ▸; Richard, 2022 ▸). Perturbation of the native state by amino acid changes may shift the relative sub-state landscape, resulting in new traits. In any case, *K*_M_ for NADH will depend on the rate constants of the individual reactions, including the off-rates of alcohol and NAD^+^, and changes in these rates can give rise to higher *K*_M_ values, as observed for NADH for the DA1472 variant (Table 2 ▸).

## 4. Concluding remarks

The N151G/L259V double substitution of FucO results in a variant with a shifted substrate scope that does not overlap with the wild-type enzyme and this variant can be viewed as a specialist aryl-aldehyde reductase. The 9000-fold shift in catalytic efficiency as calculated from the increase of the *k*_cat_/*K*_M_ values from wild-type FucO to DA1472 for the substrate phenyl­acetaldehyde (compound **3**) (Table 2 ▸) corresponds to an estimated change in transition state stabilization of 5.4 kcal mol^−1^, as calculated from equation (4) (Fersht *et al.*, 1985 ▸):

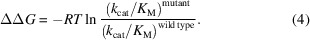

The structural data show that the two amino acid changes (N151G and L259V) do not cause any change of main-chain conformation, but do provide more space in the substrate-binding groove. Also, the geometry of the catalytic site is not affected (Figs. 6 ▸ and 7 ▸) and the enzymological data suggest that the reaction mechanism of the DA1472 variant is also the same. Thus, these two amino acid changes lead to a dramatic modification in function, illustrating the malleability of this active site by the introduction of structurally non-destructive substitutions, obtained by targeted functional selection.

## Supplementary Material

Supporting figures and tables. DOI: 10.1107/S205225252300444X/jt5067sup1.pdf

PDB reference: *E. coli* FucO mutant L259V complexed with Fe, NADH and glycerol, 7qlg

PDB reference: *E. coli* FucO mutant N151G/L259V complexed with Fe, NADH and glycerol, 7qnh

PDB reference: *E. coli* FucO mutant N151G/L259V complexed with Fe, NAD and dimethoxyphenyl acetamide, 7qlq

PDB reference: *E. coli* FucO mutant N151G/L259V complexed with Fe, NADH and dimethoxyphenyl acetamide, 7qls

## Figures and Tables

**Table 1:** Data collection, data-processing and structure refinement statistics

Dataset (cocrystallization; cryoprotectant)	D93 (L259V) (Fe^2+^, NADH; glycerol)	DA1472 (N151G/L259V) (Fe^2+^, NADH; glycerol)	DA1472 (N151G/L259V) (Fe^2+^, NAD^+^, compound 7; PEG600)	DA1472 (N151G/L259V) (Fe^2+^, NADH, compound 7; PEG600)
Data collection				
Beamline	DLS (I24)	Bruker Microstar	MAX IV, BioMAX	MAX IV, BioMAX
Detector	PILATUS 6M	PHOTON II	EIGER 16M	EIGER 16M
Wavelength (Å)	0.9999	1.54178	0.9762	0.9762
Temperature (K)	100	100	100	100

Data processing				
Space group	*P*2_1_	*P*2_1_	*P*2_1_2_1_2_1_	*P*2_1_2_1_2_1_
*a*, *b*, *c* (Å)	69.78, 55.36, 107.69	69.54. 54.65, 106.56	61.29, 85.45, 137.23	61.26, 86.43, 137.83
α, β, γ (°)	90.00, 103.67, 90.00	90.00 103.41 90.00	90.00, 90.00, 90.00	90.00, 90.00, 90.00
Data-processing software	*XDS*, *AIMLESS*	*PROTEUM3*, *AIMLESS*	*DIALS*, *AIMLESS*	*XDS*, *AIMLESS*
Resolution (Å)†	2.00 (2.05–2.00)	2.20 (2.27–2.20)	2.60 (2.72–2.60)	2.40 (2.49–2.40)
*R*_pim_ (%)	15.3 (75.2)	8.2 (29.2)	8.2 (73.9)	3.4 (34.9)
*CC*_1/2_ (%)	97.1 (41.4)	98.8 (85.0)	99.5 (52.2)	99.9 (77.6)
*I*/σ(*I*)	7.4 (2.4)	5.3 (1.9)	8.3 (2.1)	14.1 (2.5)
Completeness	98.9 (99.2)	99.8 (99.8)	100.0 (100.0)	100.0 (100.0)
Redundancy	3.1 (3.2)	1.9 (1.9)	13.8 (14.2)	13.3 (13.7)
Observed reflections	168944 (12972)	76750 (6744)	317352 (38823)	391704 (41478)
Unique reflections	53704 (3990)	39813 (3466)	22909 (2734)	29415 (3029)
Wilson *B* factor (Å^2^)	18.4	14.1	48.8	54.4

Refinement statistics				
Resolution	51.66–2.00	51.88–2.20	56.02–2.60	73.33–2.40
*R*_work_ (%)	18.6	20.9	18.7	19.7
*R*_free_ (%)	22.7	23.2	23.3	23.0
No. of used reflections	51088	37842	21742	27888
Total no. of non-hydrogen atoms	6045	5956	5858	5871
No. of waters	241	176	54	77
Average *B* factor				
Protein (A/B chains) (Å^2^)	21.5 / 22.0	23.8 / 23.3	55.0 / 59.5	65.2 / 63.9
Active site ligands‡ (average of A and B chains) (Å^2^)	25.2 (NADH), 41.9 (Fe^2+^)	23.8 (NADH), 54.7 (Fe^2+^)	83.2 (**7**), 51.3 (ADPR), 44.2 (Fe^2+^)	75.7 (**7**), 55.7 (ADPR), 46.9 (Fe^2+^)
Waters (Å^2^)	24.6	20.6	41.3	50.5
RMS deviation				
Bond lengths (Å)	0.0057	0.0078	0.0038	0.0040
Bond angles (°)	1.4	1.5	1.3	1.3
Ramachandran plot§				
Favored region (%)	97.9	96.5	97.2	97.5
Allowed region (%)	1.7	3.0	2.1	2.0
Outlier region (%)	0.4	0.5	0.7	0.5
PDB entry	7qlg	7qnh	7qlq	7qls

† Values given in parentheses are for the highest-resolution shell.

‡ ADPR refers to ADP-ribose; **7** is compound **7** (3,4-di­meth­oxy­phenyl­acetamide).

§ Williams *et al.* (2018 ▸).

**Table 2:** Steady-state kinetic parameters of aldehyde reduction and NADH oxidation by wild-type FucO and by the A5 (N151G), D93 (L259V) and DA1472 (N151G/L259V) FucO variants *K*_IS_ is the equilibrium dissociation constant of the non-productively bound aldehyde substrate (see main text, Fig. S2). Further details are described in the Materials and methods[Sec sec2].

Enzyme	Varied substrate	*k*_cat_ (s^−1^)	*K*_M_ (m*M*)	*k*_cat_/*K*_M_ (s^−1^ m*M* ^−1^)	*K*_IS_ (m*M*)
Wild-type	NADH	15±0.2†	0.020±0.001†	970±40†	
A5	NADH	2.8±0.1‡	0.029±0.006‡	96±20‡	
D93	NADH	33±2‡	0.069±0.01‡	470±60‡	
DA1472	NADH	51±2§	0.67±0.04§	75±2§	
Wild-type	**1**	26±2	2.1±0.5	12±2	
A5	**1**	3.3±0.2	12±2	0.27±0.02	
D93	**1**	22±0.9	3.1±0.5	6.9±0.9	
DA1472	**1**	9.5±0.5¶	14±3	0.66±0.1¶	
Wild-type	**2**	12±0.4	0.36±0.05	34±4	
A5	**2**	13±2	37±9	0.35±0.04	
D93	**2**	55±3	0.39±0.07	140±20	
DA1472	**2**	>8.7¶ ††	>10††	0.44±0.1¶	
Wild-type	**3**	0.023±0.01‡‡	23±10‡‡	0.0011±0.0002‡‡	
A5	**3**	2.1±0.1‡‡	12±0.7‡‡	0.17±0.005‡‡	
D93	**3**	0.62±0.1‡‡	48±12‡‡	0.013±0.8‡‡	
DA1472	**3**	28±6¶ †	2.7±0.8†	10±4¶ †	5.5±2†
DA1472	**4**	8.6±0.4¶ †	0.13±0.02†	67±10¶ †	14±4†
DA1472	**5**	16±2¶ †	0.23±0.05†	70±20¶ †	1.1±0.3†
DA1472	**6**	11±0.7¶ †	0.42±0.05†	26±3¶ †	12±6†

† Blikstad & Widersten (2010 ▸); determined in the presence of 10 m*M* of compound **1**.

‡ Determined in the presence of 50 m*M* of compound **1**.

§ Determined in the presence of 30 m*M* of compound **1**.

¶ Underestimated due to the relatively high *K*_M_^NADH^.

†† Unable to reach enzyme saturation within accessible substrate concentration. The > sign indicates the lower range for the parameter value, limited by the concentration range used.

‡‡ Blikstad *et al.* (2013 ▸).

§§ Determined after fitting a model of reversible substrate inhibition [equation (3)].
