# Supplementary material for: Crystal structures and kinetic studies of a laboratory evolved aldehyde reductase explain the dramatic shift of its new substrate specificity
Source: IUCrJ. 2023 Jun 2;10(Pt 4):437–47. doi: 10.1107/S205225252300444X (PMC10324492; doi:10.1107/S205225252300444X)
Supplement: Supplementary file 1 [file m-10-00437-sup1.pdf]

IUCrJ

Volume 10 (2023)

Supporting information for article:

Crystal structures and kinetic studies of a laboratory evolved aldehyde reductase explain the dramatic shift of its new substrate specificity

Shruthi Sridhar, Alberto Zavarise, Tiila-Riikka Kiema, Subhadra Dalwani, Tor Eriksson, Yannick Hajee, Thilak Reddy Enugala, Rik K. Wierenga and Mikael Widersten

Index

Supporting Tables

Table S1. Crystallization and crystal treatment protocols... S2

Table S2. Medium viscosity effects on reaction rates of the DA1472 variant…….................... S3

Supporting Figures

Figure S1. Electron density 2Fo-Fc omit maps.........…………………...................................... S4

Figure S2. Kinetic data of the DA1472 variant........................…….. S5

Figure S3. pH dependency of the reduction reaction of the DA1472 variant.............................. S6

Table S1. Crystallization and crystal treatment protocols

**Data set (cocrystallization; cryoprotectant)**	D93 (L259V) (with Fe^{2+}, NADH; glycerol)	DA1472 (N151G/L259V) (with Fe^{2+}, NADH; glycerol)	DA1472 (N151G/259V) (with Fe^{2+}, NAD^+, substrate analog (compound **7**); PEG600)	DA1472 (N151G/L259V) (with Fe^{2+}, NADH, substrate analog (compound **7**); PEG600)
**PDB entry**	7QLG	7QNH	7QLQ	7QLS
Protein buffer	10.03 mg/ml dissolved in 20 mM Tris-HCl, pH 7.5 that was supplemented with 0.5 mM $FeCl_2$ and 10 mM NADH and incubated for 10 min at room temperature.	9.25 mg/ml dissolved in 20 mM Tris-HCl, pH 7.5 that was supplemented with 0.5 mM $FeCl_2$ and 10 mM NADH and incubated for 10 min at room temperature.	9.25 mg/ml dissolved in 20 mM Tris-HCl, pH 7.5 that was supplemented with 1 mM substrate analog 0.5 mM $FeCl_2$ and 10 mM NAD^+ and incubated for 10 min at room temperature.	9.25 mg/ml dissolved in 20 mM Tris-HCl, pH 7.5 that was supplemented with 1 mM substrate analog 0.5 mM $FeCl_2$ and 10 mM NADH and incubated for 10 min at room temperature.
Well solution buffer	50.64 mM sodium acetate trihydrate, pH 4.5; 15 % (w/v) PEG 3350; 200 mM sodium formate.	50.64 mM sodium acetate trihydrate, pH 4.5; 15 % (w/v) PEG 3350; 200 mM sodium formate.	0.1 M bis-tris pH 5.5; 20 % (w/v) PEG 6000; 0.2 M sodium formate.	0.1 M sodium acetate pH 4.5; 20 % (w/v) PEG 8000; 0.2 M sodium formate.
Drop size	0.4 µL + 0.4 µL	0.4 µL + 0.4 µL	0.4 µL + 0.4 µL	0.4 µL + 0.4 µL
Crystal soaking protocol (time, composition)[a]	The crystal was transferred in 1 µL well solution, supplemented with 10 mM NADH and diluted with glycerol to a final concentration of 30 % (v/v), for 1 min.	The crystal was transferred in 1 µL well solution, supplemented with10 mM NADH, and diluted with glycerol to a final concentration of 30 % (v/v), for 1 min.	The crystal was transferred in 1 µL well solution, supplemented with10 mM NAD^+ + 1 mM compound **7** and diluted with PEG600 to a final concentration of 20 % (w/v), for 1 min.	The crystal was transferred in 1 µL well solution, supplemented with10 mM NADH + 1 mM compound **7** and diluted with PEG600 to a final concentration of 20 % (w/v), for 1 min.
Sample name (in IceBear[b])	FUCO_96ehE08d2c5	FUCO_96ekE08d1c1	FUCO_96j2G10d2c2	FUCO_96j1F06d3c1

[a] Each crystal was cryoprotected at the end of the crystal treatment protocol by immersing in liquid nitrogen.

[b] Daniel, E., Maksimainen, M. M., Smith, N., Ratas, V., Biterova, E., Murthy, S. N., Rahman, M. T., Kiema, T.-R., Sridhar, S., Cordara, G., Dalwani, S., Venkatesan, R., Prilusky, J., Dym, O., Lehtio, L., Koski, M.K., Ashton, A.W., Sussman, J. L. & Wierenga, R. K. (2021) Acta Cryst. D**77**, 151-163.

Table S2. Medium viscosity effects on reaction rates of the DA1472 variant

Enzyme	Substrate	η_{rel}	k_{cat} (s^{-1})	k_{cat}/K_M (s^{-1} mM^{-1})	k_{cat} dependency on medium viscosity[a]	k_{cat}/K_M dependency on medium viscosity[a]
DA1472	compound **5**	1	8.0±0.005	80±6		
	compound **5**	1.5	5.6±0.006	43±7	1.2±0.1	1.0±0.2
	compound **5**	2.8	2.5±0.002	27±2		

[a] The slopes are calculated from the linear fits shown in **Fig. S2C**.

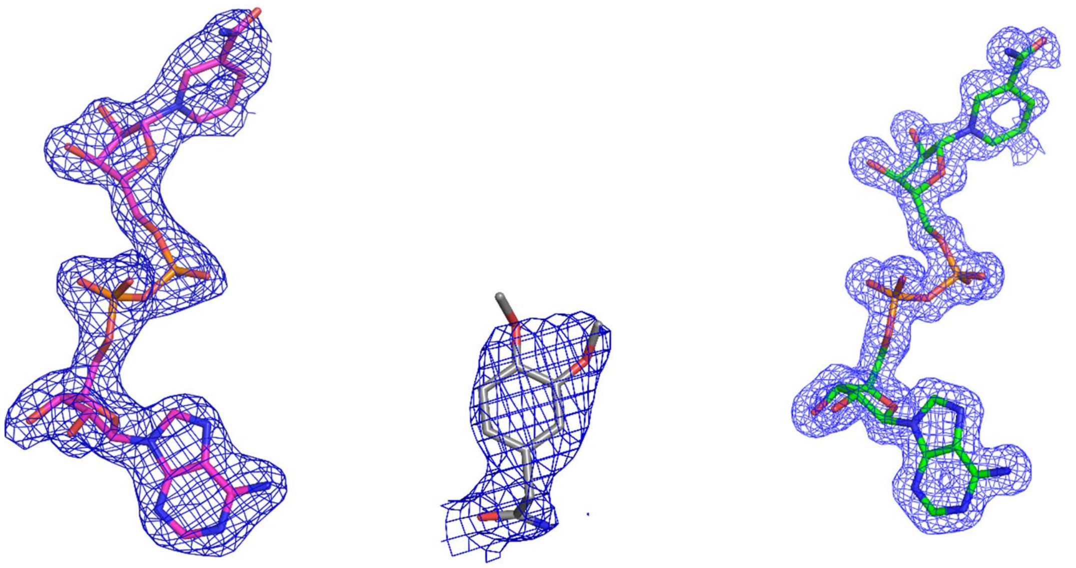

(a) (b) (c)

Figure S1. Electron density 2Fo-Fc omit maps, calculated using models in which the specified ligand was omitted from the model. The maps have been contoured at 1 sigma. (a) The DA1472 structure (PDB entry 7QNH) in which NADH of chain B was omitted. (b) The DA1472 structure (PDB entry 7QLS) in which the bound substrate analog (compound **7**, 3,4-dimethoxyphenylacetamide) of chain B was omitted from the model. (c) The D47 structure (PDB entry 7R0P) in which the bound NAD^+ of chain A was omitted.

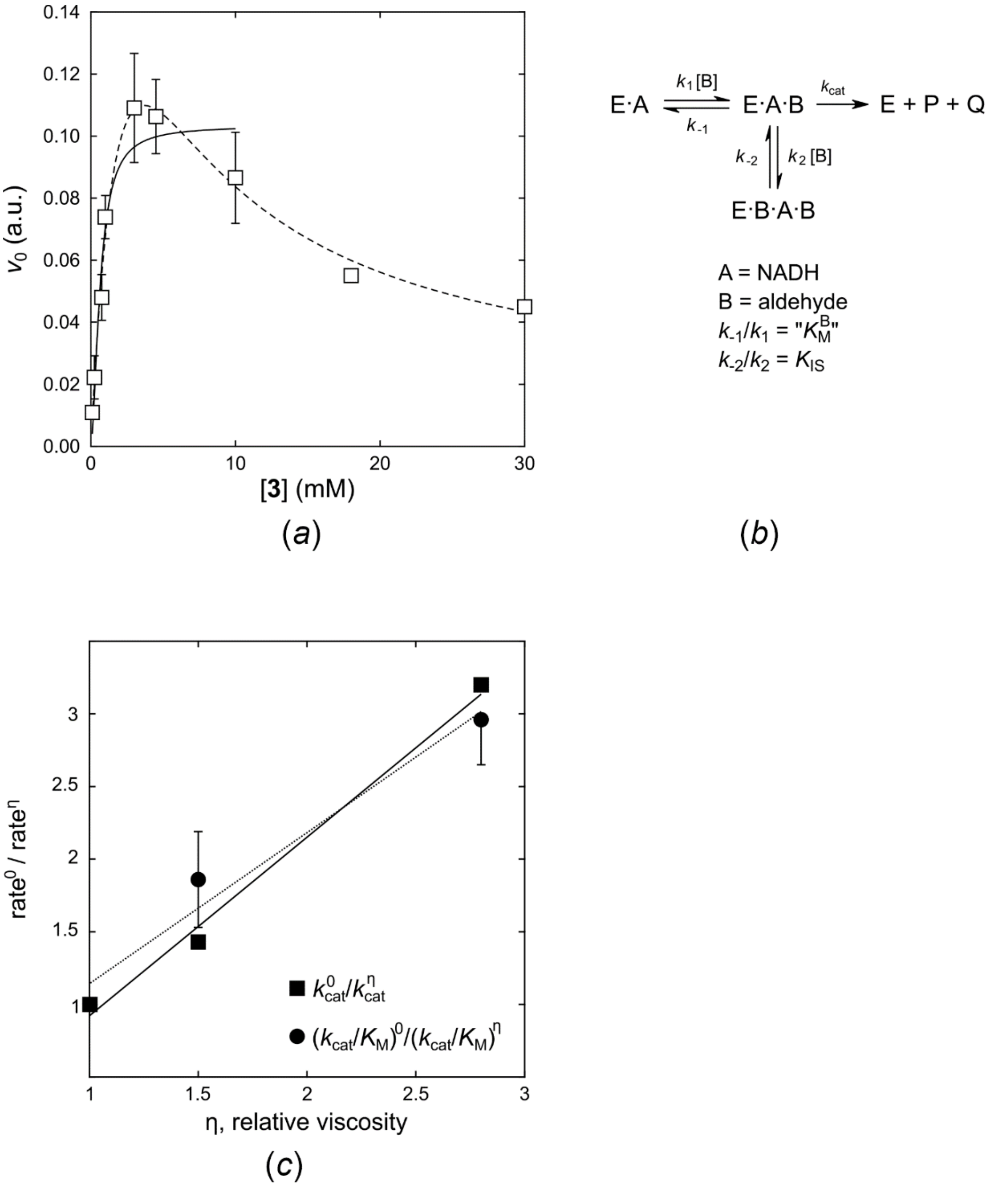

Figure S2. Kinetic data of the DA1472 variant. (a) Model fitting to steady state data of DA1472 catalyzed reduction of compound **3**. Solid line: Michaelis-Menten model, and dashed line: a model including an additional step of an alternative aldehyde binding mode resulting in a nonproductive dead-end complex. Units of activity is ΔA_{340}/min. The model used is shown in (b). Extracted parameter values (Eq. 3) are given in the main text. (c) Influence of medium viscosity on steady state rate constants k_{cat} (■, solid line) and k_{cat}/K_M (●, dotted line) in the

DA1472 catalyzed reduction of compound **5**. The unit dependencies (**Table S2**) of both parameters suggest product release to be rate limiting for turnover.

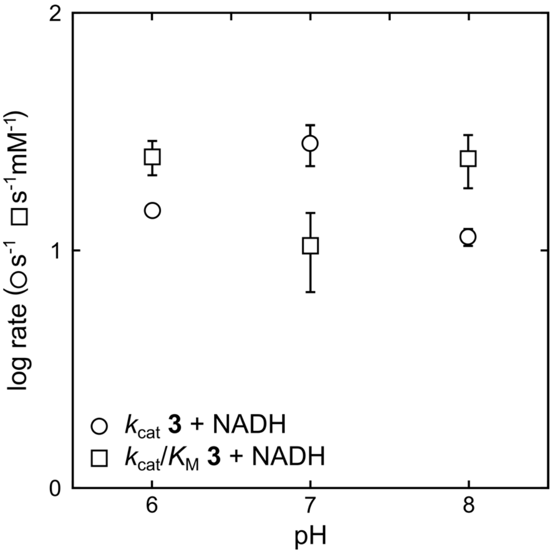

Figure S3. pH dependency of the reduction reaction of the DA1472 variant. The pH dependency of the k_{cat} and k_{cat}/K_M of the DA1472 catalyzed reduction of compound **3** in the presence of 0.4 mM NADH.
